# Exploring the Potential of Receptor Silencing in the Tumor Microenvironment by RNA Interference

**DOI:** 10.3390/biom16020315

**Published:** 2026-02-17

**Authors:** Karina Mayumi Tani Bezerra de Melo, Beatriz Mendonça Alves Bandeira, Pedro Vinícius Silva Novis, Micaela Evellin dos Santos Silva, Ingrid Andrêssa de Moura, Antonio Carlos de Freitas, Anna Jéssica Duarte Silva

**Affiliations:** Laboratory of Molecular Studies and Experimental Therapy—LEMTE, Department of Genetics, Federal University of Pernambuco, Avenida da Engenharia S/N, Recife 50740-600, Pernambuco, Brazil; karina.mayumi@ufpe.br (K.M.T.B.d.M.); beatriz.mendonca@ufpe.br (B.M.A.B.); pedro.novis@ufpe.br (P.V.S.N.); micaela.evellin@ufpe.br (M.E.d.S.S.); ingrid.andressa@ufpe.br (I.A.d.M.)

**Keywords:** gene therapy, immune checkpoint, growth factor receptors, miRNA, siRNA, shRNA

## Abstract

Cancer is a heterogeneous disease caused by genetic and epigenetic factors, leading to alterations in signaling pathways and regulatory processes. Overall, the more commonly employed conventional treatments present side effects and resistance. Due to the diverse cellular composition of the tumor microenvironment, inhibition of cell communication by RNA interference (RNAi) has emerged as a strategy to regulate the expression of receptors linked to carcinogenesis. This review examines RNAi-mediated receptor silencing as a strategy to modify the tumor microenvironment, primarily in tumor cells, enhancing its vulnerability to immune cell destruction and reducing resistance to conventional therapies. In the tumor microenvironment, the silencing of immune checkpoints like PD-1 and CTLA-4 has demonstrated the ability to restore T cell function and enhance the efficacy of adoptive cell therapies. Additionally, the targeting of G protein-coupled receptors, including CXCR4, CCR5, and A2aR, as well as growth factor receptors such as VEGFR and EGFR, and interleukin receptors, interferes with pathways that are critical for tumor promotion, resulting in diminished angiogenesis, metastasis, and immunosuppression. These strategies utilize advanced delivery systems, including nanoparticles and exosomes, and show that silencing multiple targets can produce more effective antitumor outcomes than single-target methods, underscoring the significant potential of RNA interference in cancer treatment.

## 1. Introduction

Cancer is a complex condition resulting from the dysregulation of multiple cellular pathways that are essential for the maintenance of homeostasis, which results, for example, in uncontrolled proliferation and alteration of the cell cycle [1]. Despite current therapeutic strategies, many patients still face tumor recurrence and resistance to conventional treatments due to the heterogeneous nature of the disease [2]. This heterogeneity is closely linked to the influence of the tumor microenvironment, which plays a role in cancer progression and resistance [3].

The tumor microenvironment (TME) is composed of a variety of cells, an extracellular matrix, and physicochemical factors [4,5]. The interaction between these components leads to the modulation of the microenvironment through various signals of angiogenesis, cell growth, and apoptosis inhibition, which are intrinsically related to inflammatory processes and immune evasion [6]. These elements restrict traditional treatments based on chemotherapy and radiotherapy, due to acquired resistance, thus providing the opportunity for the development of new immunotherapies aimed at modulating the TME [7]. In addition, immunotherapy often elicits more durable responses by modulating the patient’s own immune cells. This mechanism contrasts with that of conventional therapies, whose targets are the tumor cells that, due to their high genetic instability, have a strong predisposition to develop mechanisms of resistance to traditional drugs [8].

In this context, gene therapy represents a promising therapeutic strategy that may enhance and potentially surpass the limitations of conventional medicines [9,10]. Gene therapy uses genes to help fight diseases through gene addition, editing, or expression modulation [11]. In cancer, gene silencing, or expression modulation, has the potential to limit the growth and survival capacity of tumor cells while also modulating the tumor microenvironment. Inhibiting the expression of genes that code for receptors linked to pro-tumor traits offers a viable approach for disease therapy [12]. Reducing the expression of receptors that facilitate tumor cell proliferation can effectively limit tumor growth. The repression of genes influencing the polarization of immune cells towards a pro-tumoral state may reverse the immunosuppressive environment, thereby enhancing the immune system’s ability to combat cancer [13]. This method directly disrupts and modifies the tumor microenvironment, rendering it less conducive to development and resistance [14].

Therefore, this review aims to examine the role of the tumor microenvironment by discussing how receptor silencing strategies, predominantly assessed in neoplastic cells, may indirectly influence TME-associated mediators and contribute to cancer treatment. Furthermore, we address the clinical and therapeutic applications of these approaches while acknowledging current limitations and outlining future perspectives for RNA interference-based therapeutics.

## 2. RNA Interference

Discovered in 1998, RNAi is a post-transcriptional silencing mechanism that can modulate the expression of a target gene by inducing the degradation of messenger RNA (mRNA) or blocking translation, resulting in the reduction in the correspondent protein [15,16]. In recent years, RNAi has been gaining prominence, being applied in different areas of biotechnology, such as the development of pesticides for the agricultural sector and drugs for medicine [17].

**Figure 1:**
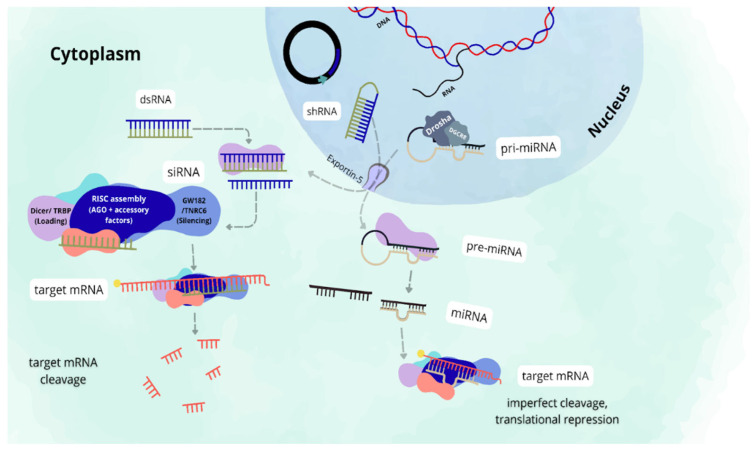
Main types of RNAi and gene regulation mechanisms.

Despite the common strategy, there are three main types of RNAi, which differ in structure, origin, and gene regulation mechanism (Figure 1) [18,19]. Both small interfering RNA (siRNA) and microRNA (miRNA) are produced from double-stranded RNA (dsRNA), but they differ in their origin. While siRNA can be derived from viral genomes, bacterial DNA, or introduced as a synthetic molecule, miRNA is of an endogenous origin [20]. In turn, short hairpin RNA (shRNA) is an artificial construct whose sequence needs to be inserted into the genome of the target cell through vectors. After transcription, the shRNA transcript is processed by the cellular machinery to generate a functional siRNA [20].

The processing of shRNA begins with its transcription in the nucleus, where it is synthesized as a precursor loop molecule (pre-shRNA) [20]. Exportin-5 sends this molecule to the cytoplasm, where the enzyme Dicer cuts it into a small double-stranded siRNA [20,21]. This siRNA is incorporated into the RNA-induced silencing complex (RISC), a multiprotein complex centered on Argonaute (AGO) proteins, particularly AGO2, which mediates target mRNA recognition and cleavage [18,20,21]. RISC assembly is a multistep process assisted by accessory factors, including Dicer and TRBP during small RNA loading, and molecular chaperones such as Hsp90/Hsc70 that promote Argonaute activation [22]. Downstream gene silencing is further supported by GW182/TNRC6 proteins, which link RISC to translational repression and mRNA decay machinery [23].

In contrast, miRNA often binds to regions of mRNA with imperfect complementarity, which allows it to regulate a vast gene network by repressing translation or inducing the degradation of multiple mRNAs [24,25]. Regarding its processing, the endogenous miRNA begins as a primary transcript (pri-miRNA) that is processed in the nucleus by the Drosha-DGCR8 complex, resulting in the pre-miRNA [24]. This, in turn, is exported to the cytoplasm by Exportin-5 and cleaved by Dicer, where the guide strand of the mature miRNA is then incorporated into RISC [18].

Currently, there are eight products approved by the Food and Drug Administration (FDA) that use RNAi for the treatment of diseases. Patisiran (Onpattro) was the first product with RNAi technology to be approved by the FDA in 2018, and it was a milestone for the approval of other RNAi-based products [16]. It targets the mutant transthyretin (TTR) protein in hereditary transthyretin amyloidosis (ATTRv). Following this milestone, the FDA has approved a new product annually until 2023 [21]. These products are intended to treat metabolic and liver diseases, but there are various clinical trials underway for different diseases, including cancer (Table 1) [16].

Despite notable progress in cancer treatment, traditional therapies continue to demonstrate low specificity, considerable systemic toxicity, and the emergence of tumor resistance. The identified limitations undermine long-term therapeutic efficacy and adversely affect patients’ quality of life [26]. Similarly, targeted therapies, although more directed at specific molecular alterations, also face challenges. These are related to tumor heterogeneity, the emergence of cellular escape mechanisms, and the limitation of efficacy to restricted subgroups of patients carrying certain genetic mutations [26,27].

In targeted therapies based on monoclonal antibodies, such as immune checkpoint inhibitors, lasting clinical responses are observed only in a portion of patients. In addition, they are linked to the occurrence of immune-mediated adverse events, high production costs, and difficulties related to tumor penetration and the immunogenicity of these agents [28]. FDA-approved monoclonal antibodies, such as Rituximab and Pembrolizumab, face cases of drug resistance generated by signaling, problems of accessibility to the tumor site, and the heterogeneity of the TME [29].

The use of RNAi in patients with early-stage tumors (I-III) aims to modulate genes that are related to tumor growth and progression [30]. In the oncological context, there are some therapies such as siG12D LODER, which was developed for silencing the KRAS G12D mutation in pancreatic ductal adenocarcinoma, and siRNA-EphA2-DOPC, aimed at blocking the EPHA2 receptor in advanced solid tumors. They illustrate the potential of RNAi to directly interfere in tumor progression pathways, such as the RAF-MEK-ERK pathway and the PI3K-AKT-mTOR pathway [1,31,32]. STP705 and STP707 are more recent strategies, which promote the concomitant silencing of TGFB1 and PTGS2 and highlight approaches aimed at modulating the TME, with the goal of reducing immunosuppression and increasing the antitumor response [1]. As a result, RNAi-based approaches have reached a level of technological maturity that is compatible with clinical applications, presenting themselves as a versatile and promising therapeutic platform for different diseases, including tumors characterized by high biological complexity [1].

Furthermore, RNAi-based therapy, when used in a standalone regimen, exhibits high specificity in gene silencing, allowing for direct inhibition of molecular targets associated with tumor progression, drug resistance, and immune system evasion [33]. However, evidence shows that combining RNAi with chemotherapy, immunotherapy, or targeted therapies can result in synergistic effects, either by sensitizing tumor cells, modulating the tumor microenvironment, or overcoming previously established molecular resistance mechanisms [16]. Furthermore, combinatorial approaches involving RNAi have shown promise in reducing the required doses of conventional therapeutic agents, contributing to a decrease in adverse effects and an improvement in the safety profile of treatments [34].

Although RNAi studies have shown themselves to be an alternative for silencing a specific gene, some important observations are necessary for the construction of an RNAi (siRNA/shRNA). These observations aim to ensure the best possible effectiveness of silencing, targeting, and patient safety after its administration.

### 2.1. Development and Expression of RNAi

The RNAi sequence must be specific to the gene that one wishes to silence: that is, it must not impact the expression of other genes, avoiding off-target effects due to homologous sequences [35]. Therefore, in silico tools are used to analyze candidate sequences in the target genome and evaluate their possible effects [35]. In addition to homologous sequences, it is important to consider the structure and conformation of the RNA molecule, considering the RNAi binding region in the molecule, whether it is a more internal or external region, and its affinity with mRNA.

Also, in relation to reducing off-target effects and also to improving its efficiency and stability, some chemical modifications can be performed, in isolation or in combination [36]. Modifications to the oxygen in the non-binding phosphate region by a sulfate, known as phosphorothioate (PS), can improve RNAi biodistribution, and decrease its degradation by nucleases [36,37]. Modifications to the 2′-OH region, through the incorporation of 2′-O-methyl or 2′-fluoro, have proven efficient in improving thermal stability and double-strand RNA stability [37].

Regarding RNAi expression in cells, it is necessary to construct a plasmid that can express RNAi in a stable and safe manner. The most commonly used promoters for RNAi expression are those that utilize polymerase III for transcription, such as U6 and H1 [38]. Between these two, U6 is the most widely used, due to the better performance in silencing genes from RNAi sequences subjected to this promoter [39,40,41,42]. Flanking sequences, such as miR30, are used to help stabilize RNAi and also to reduce the toxicity of this molecule [43].

There are studies that aim to silence more than one gene in order to obtain better results in reversing the studied disease. One of the ways to perform this silencing is by constructing plasmids that co-express two or more RNAi, called combinatorial RNAi (coRNAi) [30]. Estrin et al. (2018) used coRNAi to inhibit genes related to influenza A virus replication, which may include, for example, mutant strains [44]. In this study, different combinations were tested, and the combination with the best silencing and low toxicity was chosen [44]. In addition to the toxicity capacity depending on the type of combination, it is important to highlight the number of RNAi that can be expressed on the same plasmid. It was reported that more than 5 shRNAs in the same plasmid provoked a decrease in efficacy [45].

To circumvent this issue, more than one plasmid for different RNAi sequences can be employed. This effect was observed in a study where the mutant p53 gene and tumor necrosis factor (TNF) were silenced, resulting in the induction of apoptosis in triple-negative breast cancer cells [46]. Overall, it is still important that studies involving multiple RNAi address parameters including off-target effects, stability, and expression of the insertion of multiple plasmids.

### 2.2. RNA Interference: Limitations and Delivery Strategies

Despite the emergence of RNAi as a therapeutic strategy, the approach presents some physicochemical and biological obstacles, facing multiple barriers imposed by the TME that can compromise its efficacy [47]. The TME is characterized by abnormal vasculature, dense extracellular matrix, acidic pH, hypoxia, and heterogeneous stromal and immune cell populations, all of which collectively limit RNAi stability, penetration, and cellular uptake [48]. One of the main problems is that RNAi is unstable in the body, which makes it more likely to be broken down by nucleases. This leads to renal clearance, which makes it less available and less effective as a treatment [49,50]. Also, the degradation of RNAi can activate immune responses by recognizing it as foreign, thereby triggering an undesired innate immune response within the immune cells present in the TME [47,51]. In this sense, some studies have promoted chemical modifications of RNAi, incorporating 2′-O-methyl and 2′-fluoro phosphorothioate, which demonstrated increased resistance to enzymatic degradation and consequently reduced immunogenicity [52,53].

As it is an anionic molecule and carries a negative charge, RNAi suffers electrostatic repulsion by the plasma membrane, preventing its spontaneous entry into tumor and stromal cells within the TME [54,55]. To circumvent this, Liu et al. (2022) used cationic liposomes to coat RNAi, while Zhu et al. (2022) developed a conjugate of RNAi and a cationic cholesterol derivative (CEL); both strategies achieved effective gene silencing and overcame the electrostatic barrier in tumor-associated cells [56,57].

However, even when it manages to enter cancer cells, some intracellular barriers hinder the effective delivery of RNAi within the hostile intracellular environment of tumor cells. After internalization, the RNAi can be encapsulated by endosomes and fail to reach the target and form the RISC, either by diversion through intracellular recycling pathways or by degradation [58,59,60]. Even when it escapes from the endosomes, there are risks of degradation by nucleases present in the cytosol or being carried by non-functional compartments, such as activated stress granules. Taken together, these factors can reduce the functional portion of the available RNAi and limit effective gene silencing in the TME [47,61].

To overcome these hurdles, diverse strategies have been developed. For instance, Cheng et al. (2020) used lipid nanoparticles with ionizable lipids to facilitate endosomal escape into the cytosol, while Chernikov et al. (2024) functionalized particles via direct conjugation with membrane ligands (e.g., cholesterol-RNAi) [62,63]. Both aim to improve cellular internalization and lipid affinity in tumor-resident cells [62,63]. Other prominent approaches include polymeric particles and microorganisms, such as yeasts aimed at improving RNAi delivery within the TME [64,65].

#### 2.2.1. Lipid Particles

Lipid nanoparticles (LNPs) emerge as the main non-viral platform with the potential to overcome the challenges related to the efficient delivery of RNAi to target cells, facilitating cellular internalization and promoting endosomal escape in both tumor and immune cells [66,67]. These particles form a system that allows for modulation of physicochemical and biological properties, overcoming the barriers encountered by RNAi in the TME and optimizing its therapeutic effect, and can be composed of ionizable or structural lipids [68,69].

Ionizable lipids are explored for this application because they remain electrically neutral at a physiological pH, which reduces the chances of systemic toxicity, RNAi degradation, and nonspecific interactions. In acidic environments, they acquire a positive charge, allowing interaction with the membrane and the release of RNAi into the cytosol [70]. In the study by Lin et al. (2024), for example, the charge of the RNAi was optimized using a formulation of DLin-MC3-DMA, an ionizable lipid with mPEG-b-PLGA, a cationic lipid [71].

Another strategy used is the functionalization of the LNP surface with specific ligands, such as peptides or antibodies, allowing for greater targeting of RNAi to specific tissues, reducing side effects, and increasing its therapeutic effect [72,73]. Nai et al. (2022) developed a hybrid platform where RNAi coated with a macrophage membrane and the peptide cRGD [74]. This peptide has a high-affinity ligand for αvβ3 integrins, which is overexpressed in tumor cells, allowing the release of the therapeutic agent with greater tumor specificity and increased cellular internalization [74].

As a safe, non-viral alternative for RNAi delivery, the development of cationic liposomal systems also emerges as an alternative. This approach is characterized by lipid vesicles that present a positive charge on their surface and, by forming an electrostatic complex with the RNAi, protect it from degradation and favor interaction with the plasma membrane, facilitating its internalization by endocytosis [75,76]. Sánchez-Meza et al. (2024) developed a study on cationic liposomes for RNAi delivery, targeting the E6 gene of HPV16, an oncogene important for tumor development [77]. The liposomes were developed from DOTAP (1,2-dioleoyl-3-trimethylammonium-propane), a cationic lipid, combined with DOPE (dioleoylphosphatidylethanolamine), a neutral lipid that aids in the fusion of vesicles with endosomal membranes [75,78]. The internalization of RNAi through electrostatic interactions leads to the release of RNAi into the cytosol due to endosomal destabilization promoted by DOPE, associating with the RISC and promoting E6 silencing.

#### 2.2.2. Polymeric Particles

Still in the context of nanoparticles, polymeric particles emerge as a strategy to optimize and overcome the obstacles faced by RNAi in the TME, mainly due to their structural versatility, being able to meet different delivery needs [79]. These systems are constructed from synthetic or natural polymers, such as poly(lactic-co-glycolic acid) (PLGA), polyethyleneimine (PEI), chitosan, and others. They allow the projection of particles that protect RNAi from degradation by nucleases, facilitate cellular internalization, and direct it to the cytosol in tumor and stromal cells [53,79,80].

W. Zhang et al. (2019) developed a chitosan nanoparticle modified with hyaluronic acid to transport RNAi targeting the B-Cell Lymphoma 2 (BCL2) gene, with a focus on lung cancer [81]. The nanoparticles containing this modification exhibited higher cellular internalization compared to unmodified nanoparticles or free RNAi [81]. Moreover, there was a significant reduction in BCL2 expression in the target cells, resulting in tumor growth retardation through modulation of the TME [81].

Furthermore, hybrid systems combining polymeric particles with chemotherapeutic or immunomodulatory drugs have also demonstrated efficacy in optimizing RNAi. J. Zhao et al. (2023), for example, developed hybrid nanoparticles with PLGA and chitosan to coencapsulate doxorubicin (DOX), a chemotherapeutic agent, and RNAi targeting the BIRC5 gene (Baculoviral IAP Repeat Containing 5), which inhibits apoptosis [82]. The study observed the simultaneous release of DOX and RNAi, which occurred from the controlled degradation of PLGA after the internalization of the nanoparticles. This process led to the silencing of BIRC5 and an increase in tumor apoptosis, suggesting the potential of hybrid strategies to produce synergistic effects and combat tumor resistance [82].

In addition to lipid and polymeric particles, there are hybrid constructs that utilize polymers and lipids. For example, X. Xu et al. (2022) developed a system called “Matryoshka” that carries an RNAi targeting the Nogo-B receptor and carries the drug Paclitaxel (PTX) [83]. This system is composed of GE11, a polypeptide that targets endothelial growth factor receptor (EGFR), which is highly present in cells that are resistant to the drug PTX, and polyethylene glycol (PEG), which serves as a protective layer that aids in blood circulation and protect the internal content. A layer of PEI, which helps in the transport of siRNA, increases the efficiency of its silencing. Besides mesoporous organosilica nanoparticles (MONs), which serve as a carrier and provide controlled drug release, and polymers coupled with MONs that help with biocompatibility [83], by presenting several layers that are gradually degraded to then release their contents, this system showed excellent results due to its diverse structure that allows for the recognition of resistant cells, the stability of its internal content, and its release [83].

Despite the results in the experimental studies, there are some limitations to be addressed in the use of NPs as carriers, such as the functionalization of NPs through conjugation with various molecules, like PEG, which increases the particle size, potentially interfering with the delivery process [84].

#### 2.2.3. Yeast

Besides approaches based on synthetic nanoparticles, biological systems are being explored for the delivery of RNAi therapies. In this context, microorganisms that are generally recognized as safe (GRAS), such as the yeasts *Pichia pastoris* and *Saccharomyces cerevisiae*, stand out as a delivery alternative [65,85]. Due to their cell wall composition, they can act as immunomodulators through the interaction of β-glucans with pattern recognition receptors (PRRs). These receptors can induce the production of cytokines, chemokines, and other molecules that assist adaptive immunity in the fight against the pathogen [65]. Moreover, they interact with receptors that are present on antigen-presenting cells (APCs), such as Dectin-1 in macrophages and dendritic cells (DCs), facilitating their recognition and phagocytosis and favoring more specific drug delivery when these APCs are the target cell [65].

Moreover, due to their structural composition, yeasts allow for oral administration, which provides a significant logistical and clinical advantage as a non-invasive and more accepted route [86]. This route is particularly advantageous for targeting immune cells within the tumor microenvironment of gastrointestinal cancers, where oral delivery enables direct local interaction. The presence of chitin in the cell wall acts as an enhancer of gastrointestinal absorption with a pH-responsive nature, while mannoproteins and beta-glucan plays an immunostimulatory role [87]. The structure itself also provides physical protection to the therapeutic material, offering protection when it reaches the immune cells present in the mucosa, which is hardly accessed through parenteral administration [88].

The potential of yeast-based RNAi delivery is exemplified by the work of Zhang et al. (2014), who showed that silencing CD40 can directly influence the antitumor immune response [42]. They demonstrated that oral administration of recombinant S. cerevisiae can efficiently and specifically deliver shRNA targeting CD40 to intestinal dendritic cells in mice, achieving gene silencing and modulating the secretion of key cytokines such as interleukin-12 (IL-12), tumor necrosis factor α (TNF-α) and IFN-γ [42].

However, for this RNAi delivery method targeting TME modulation to be effectively incorporated into clinical practice, some aspects must be addressed. These include sufficient accumulation and targeted release of the RNAi payload within the solid tumor mass itself, and within immune cells in associated lymphoid tissues or the bloodstream [89]. Future development of yeast vectors may require structural or surface modification, such as the use of yeast capsules and the incorporation of tumor-directed peptides that are responsive to specific tumor enzymes to facilitate selective penetration and unpacking. In yeast, functionalization to increase efficacy and reduce undesirable effects can be used, but it can interfere with the insertion of nucleic acids, making it more difficult and limited, thereby decreasing the amount of nucleic acids to be presented [85]. Moreover, in the case of oral administration of yeasts, there are some challenges such as low immunity, the difficulty of production and commercialization of dry tablets [87,88].

#### 2.2.4. siRNA Conjugates

In line with lipid-based, polymer-based, and microorganism-based delivery systems, there are also strategies that seek to improve delivery efficacy without the need for encapsulation through conjugation [90]. Compared with siRNAs that are not conjugated, this strategy is structurally less complex and provides better biocompatibility, targeting, and stability [90,91].

N-acetylgalactosamine (GalNAc), for example, evaluated in different clinical trials, is being used in FDA-approved drugs (Inclisiran, Givosiran, Lumasiran, and Vutrisiran) [90,92]. Due to its interaction with the asialoglycoprotein receptor (ASGPR), this conjugate is primarily used for RNAi delivery to the liver, making it particularly interesting for targeted administration to this organ [90]. However, recent studies have shown that GalNAc can also be applied to extrahepatic tumors, in which ASGPR is expressed in cancer cells. In the study by Liu et al., 2024, GalNAc was used to transport siRNA that silences lncRNA16, which is abnormally expressed in chemotherapy-resistant non-small cell lung cancer (NSCLC), decreasing tumor growth and resistance to chemotherapy, both in vivo and in vitro [93].

RNAi can also be conjugated with lipids, such as cholesterol [91]. Due to the presence of cholesterol in the cell membrane, RNAi internalization can occur in different ways, either by endocytosis or by interaction with lipid receptors [94]. Chernikov I.V. et al. (2016) observed that the RNAi conjugated with cholesterol resulted in accumulation in hematological cells and could be used as a carrier: for example, in leukemias and lymphomas [95]. Regarding TME cells, it was seen that the use of an siRNA conjugated to a lipid was able to increase the population of CD8+ T cells, in addition to the maturation of DCs [96]. In this sense, the subsequent sections explore the tumor microenvironment, including its biological and structural components, as well as the effects of receptor silencing found in tumor cells and their surrounding environment.

In addition to understanding how RNAi is able to silence a gene, as well as its limitations and how to overcome them using delivery platforms, some points must be taken into consideration. When silencing genes with the aim of modulating TME, it is necessary to understand how this environment works and how the present cells act in combating or stimulating the carcinogenic process, allowing for the selection of a therapeutic target.

## 3. Tumor Microenvironment

### 3.1. Cellular Components

**Figure 2:**
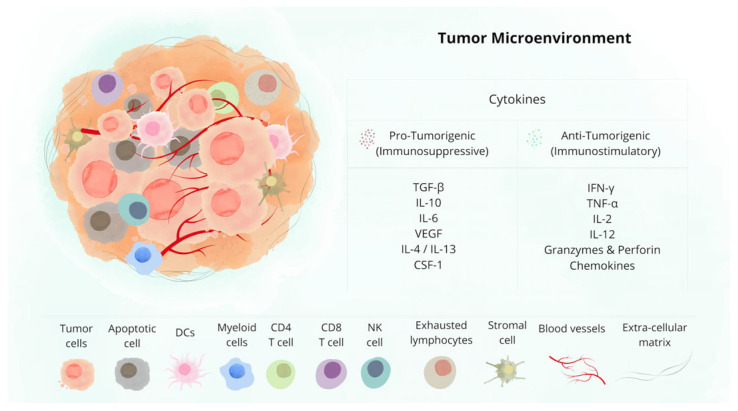
Components of tumor microenvironment.

The TME is an environment composed of malignant cells and stromal cells that are present in the extracellular matrix (Figure 2) [97]. In the TME, the interaction between tumor cells and stromal cells through cell–cell interaction promotes the modulation of the phenotype and functionality of the cells (Table 2) [98,99]. These processes are primarily tumor growth, immune system evasion, angiogenesis, and metastasis [2].

The fibroblasts that physiologically play a role in tissue repair in the tumor are considered cancer-associated fibroblasts (CAFs), the most prevalent cell type in the TME [5,100]. CAFs are part of matrix remodeling, proliferation, invasion, and immunosuppression [4,108,109].

Endothelial cells (ECs), in turn, aid to form the vascular endothelium, separating blood from tissue, providing nutrients and oxygen, and carrying immune system cells [4]. Under hypoxic conditions, ECs are stimulated to form new blood vessels from the secretion of the hypoxia-inducible factor (HIF) [102]. From these new vessels, cancer cells can reach other tissues, leading to tumor migration and, consequently, metastasis [110].

Macrophages are cells that originate mainly from monocytes present in the bloodstream [106]. Under physiological conditions, they function to eliminate pathogens and maintain homeostasis [111]. In the TME, macrophages are considered tumor-associated macrophages (TAMs), which can assume different profiles. Among these various profiles, there are M1 macrophages that have a pro-inflammatory function, acting in the elimination of tumor cells through phagocytosis, and the M2 macrophages that play an anti-inflammatory and immunosuppressive role, promoting tumor growth by reducing immune surveillance [112]. These macrophages are not fixed in a single profile and can be modulated according to the stimuli present in the TME [106].

In the TME, there are also suppressor cells that come from myeloid-derived suppressor cells (mMDSCs). They originate from hematopoietic stem cells, generating monocytes and granulocytes, which subsequently give rise to macrophages, dendritic cells, neutrophils, and eosinophils [113]. In cancer, mMDSCs are studied for their significant impact on the efficacy of immunotherapies, due to their role in TME immunosuppression [114].

Neutrophils are the first defense cells to act in inflammation and infection, as well as to activate and regulate inflammatory responses [115,116]. In the tumor, tumor-associated neutrophils (TANs) are divided into populations that are similar to macrophages, with N1 neutrophils having an anti-tumoral role and N2 neutrophils having a pro-tumoral role [115]. TANs stimulate cancer progression through the release of ROS, causing chronic inflammation, leading to DNA damage [116]. These cells also release cytokines and chemokines, which can be either anti-inflammatory or pro-inflammatory, depending on the stimulus generated by the TME [116].

DCs are a type of APC whose main function is to capture antigens, including those derived from tumors, and present them to T cells, thereby activating the immune response [117]. DCs can also present both anti-tumoral and pro-tumoral profiles, depending on the signaling of the microenvironment, which plays a role in carcinogenesis [118]. In physiological conditions, DCs are stimulated to produce inflammatory factors to destroy the tumor; however, the release of pro-tumoral mediators by the TME stimulates DCs to ignore the tumor and prevent the immune response [110,119].

When interacting with DCs, T cells undergo the process of maturation and activation, with the aim of eliminating cancer cells [120]. These cells can divide into two populations: helper T lymphocytes (CD4+ T) regulate the immune response, while cytotoxic T lymphocytes (CD8+ T) recognize and kill tumor cells [121]. Through the secretion of immunosuppressive cells and cytokines in the TME, T cells can have their functionality affected [122]. This interaction leads to the differentiation of T cells into exhausted T cells, which exhibit a super-expression of negative receptors [123]. This exhaustion also leads to a decrease in proliferation and the production of anti-tumor cytokines, favoring cancer progression [124].

Treg cells can be generated from conventional T cells and play a crucial role in tumor development and progression [107]. They are recruited to the TME through signals emitted by the tumor itself and, once activated, reduce antitumor activity, favoring immune evasion [125].

### 3.2. Extracellular Components

The extracellular matrix (ECM) plays an important role in the microenvironment, providing structural support for the tissue and nutrients for the surrounding cells. However, it also contributes to carcinogenesis by aiding in the metabolism of cancer cells, their survival, and tumor invasion into tissues (Table 3) [5,126]. It is composed of collagen, fibronectin, proteoglycans, and other glycoproteins, and each of these components communicates through adhesion receptors [5]. The remodeling of its structure through physicochemical changes affects various signaling processes that favor carcinogenesis [127].

Collagen, the main component of the ECM, plays an essential role in tissue development, providing structural support and facilitating the processes of adhesion and migration [128]. During cancer development, collagen undergoes various changes that influence the other components around it [128]. These changes can, for example, modulate the function and phenotype of immune system cells, as well as interfere with the efficacy of chemotherapy [129].

Other important components of the matrix are adhesive glycoproteins, such as fibronectin and laminin, which promote the binding between cells and the elements of the matrix itself [2]. Fibronectin plays a role in the interaction between the matrix and the cells, forming a network of fibers that is involved in cell differentiation, migration, and adhesion [110]. In tumors, they are produced mainly by macrophages and fibroblasts [2]. Laminin, on the other hand, is an important constituent of the basal membrane, along with collagen, and participates in the process of vascularization [130].

In addition to these components, the ECM is composed of enzymes called proteases. These enzymes play an important role in cell migration and invasion, as they participate in the destruction of the basal membrane and the extracellular matrix [2]. Among these enzymes, matrix metalloproteinases (MMPs) are the most studied, as they participate in the matrix remodeling process, aiding in homeostasis [131]. The degradation of the matrix, which is associated with the exposure of receptor sites that favor the interaction between its components and the release of active molecules, contributes to metastasis [131].

Extracellular vesicles, or exosomes, also play a role in the communication between tumor cells and stromal cells, as they carry biological information that participates in the initiation and progression of cancer [5]. Among the molecules carried within them are proteins, lipids, cytokines, growth factors, and micro-RNAs [5,110]. It is through these contents that angiogenesis, metastasis, and migration occur, stimulating carcinogenesis [110].

**Table 3:** Effects of tumor microenvironment extracellular components on cancer.

Extracellular Components	Alteration in Cancer	Effect of the Alteration	Reference
ECM: collagen, fibronectin, laminin	Structural and biochemical remodeling; ↑ deposition of collagens and fibronectin, ↑ MMP activity	ECM stiffening and reorganization promote invasion, metastasis, and drug resistance	[132,133]
MMPs	Increased activity (MMP-2, MMP-9)	ECM degradation and release of pro-tumoral factors, facilitating invasion and metastasis	[133]
Exosomes and Extracellular Vesicles	Increased release and altered content (miRNAs, pro-tumoral proteins)	Modulation of immunity, intercellular communication, and pre-metastatic niche formation	[134]
HIFs	Overexpression of HIF-1α and HIF-2α in hypoxic regions	Induces metabolic reprogramming (glycolysis), angiogenesis, and therapeutic resistance	[135,136]
Tumor Acidosis and Metabolism (Warburg Effect)	Increased glycolysis and lactate accumulation (↓ extracellular pH)	Suppression of antitumor immunity, activation of M2 phenotype, and increased invasiveness	[102]

Extracellular matrix (ECM), Metalloproteinases (MMPs), Metalloproteinase 2 (MMP-2), Metalloproteinase 6 (MMP-9), Micro-RNAs (mi-RNAs), Hypoxia Inducible Factor-1-Alfa (HIF-1α), Hypoxia Inducible Factor-2-Alfa (HIF-2α), M2 macrophages (M2), Increase (↑) and Decrease (↓).

### 3.3. Physical–Chemical Components

In the TME, when the rate of tumor growth exceeds the available oxygen supply, hypoxia inducible factor (HIF) is produced, stimulating the secretion of vascular endothelial growth factor (VEGF), endothelial growth factor (EGF), and the enzymes necessary for glycolysis [137]. VEGF stimulates angiogenesis, recruiting ECs, which acquire a tumor phenotype and lead to the production of abnormal vessels [135].

Furthermore, hypoxia contributes to immune tolerance and evasion of the immune system through the loss of its functions and inability to reach the tumor site [138]. It can promote the death of T cells, as well as decrease their differentiation and proliferation, and the release of some cytokines such as IL-6 [102]. In addition, in regions with low oxygen, there is a higher amount of MDSCs, TAMs, and Treg cells [135].

Due to the low amount of available oxygen, tumors alter their metabolism from oxidative phosphorylation to glycolysis, leading to the production of acidic metabolites and a decrease in the pH [135]. The change in pH in the microenvironment also has an impact on immune system cells; an acidic pH can suppress the effector T cells, decrease cytokine production, and promote the macrophages polarization to M2 in pre-clinical assays [139]. Another impact of pH is the increased production of MMPs, leading to greater ECM degradation and increased cell migration and invasion [140].

It is noteworthy that the TME is not composed solely of these elements; it is a dynamic environment that is capable of communicating and generating responses that interfere with the behavior of all its components. This communication is carried out by receptors which receive pro- and anti-tumor signals, stimulating survival, growth, and modulation of the immune response.

## 4. Receptor Silencing in the Tumor Microenvironment

### 4.1. Immune Checkpoint Silencing

**Figure 3:**
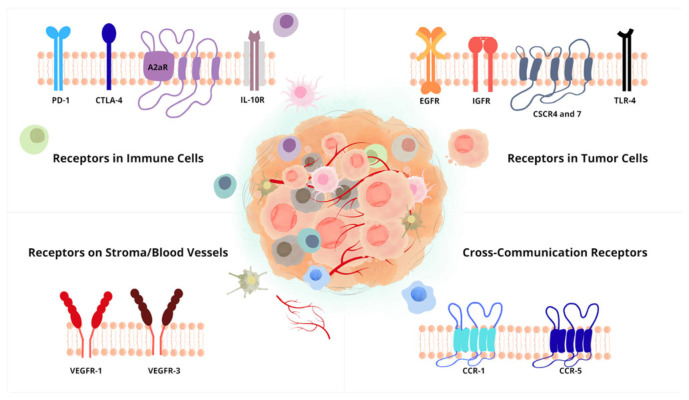
Some of the receptors in the TME.

Various receptors are present in the TME, including those related to the immune checkpoints that act as regulators of the immune response and to modulate the degree of immune activation (Figure 3) [141]. These molecules allow the immune system to respond to malignancies and infections, protecting healthy tissues from damage by preventing hyperactivation of the immune response [141]. However, in cancer, tumor cells express surface markers of immune checkpoints, leading to immune dysregulation [142].

There are approved medications that lead to the inhibition of the immune checkpoint programmed cell death-1 (PD-1), employing antibodies like Keytruda and Opdivo, for Hodgkin’s lymphoma and melanoma [143]. Similarly, the protein 4 associated with inhibitors for cytotoxic T lymphocytes (CTLA-4), Yervoy and Imjudo, is applied for metastatic melanoma and hepatocellular carcinoma [144]. Despite proving effective, these approaches have some limitations, such as their high cost and side effects, causing the search for new strategies to interfere with the action of these receptors [141].

PD-1 is a negative regulator of the immune response expressed in immune cells: mainly in CD8+ T cells [145]. In physiological conditions, this controls the hyperactivation of T cells, leading to the protection of tissues from an autoimmune response and the regulation of hemostasis [146]. In the tumor context, its interaction with its ligand, PD-L1, which is highly expressed in tumor cells, leads to the inhibition of T cell activation and cytokine production contributing to tumor evasion [147].

Unlike PD-1, CTLA-4 regulates the activity of naive T cells that are still in the lymph nodes [148]. This receptor is expressed mainly in Treg cells, but also in some effector T cells [148]. In addition to its role in controlling T cell activation, it also plays a role in inhibiting IL-2 production and modulating the development of Th2 cells [149].

Among the studies for blocking the communication between PD-1/PD-L1, a new therapeutic strategy for different tumors has emerged (Table 4) [108,115]. Studying cutaneous malignancies, Cuiffo et al. (2024) showed that the silencing of this receptor by the drug INTASYL, a self-delivering RNAi favored the increase in the infiltration of CD4+ and CD8+ T cells in the TME, and reduced the expression of PD-1 on their surface [150]. Furthermore, an increase in the secretion of anti-tumor mediators, interferon-gamma (IFN-y) and C-X-C motif chemokine ligand 10 (CXCL10), was detected [150]. Another important finding was the abscopal effect, suggesting that this treatment can also interfere with the carcinogenesis of tumors located far away from the application site [150].

Silencing of PD-1 also may be a promising alternative to increase the efficacy of adoptive immunotherapies based on the chimeric antigen receptor (CAR) [48,151]. CAR-T cell therapy consists of T cells that express CAR, aiding in the recognition of tumor cells through targeting via tumor antigens, helping in the recognition and reduction in tumor growth [152].

In the study by Zhou et al., 2021, PD-1 silencing was performed in CAR-T cells, aiming to reduce tumor immunosuppression and increase the efficacy of adoptive therapy in prostate cancer and leukemia cells [143]. Other observations included a higher proliferation of CAR-T cells and an improvement in survival (Table 4) [143]. The silencing of PD-1, through shRNA, in CAR-T can combat the exhaustion of T cells in the microenvironment [143].

**Table 4:** Studies with receptor silencing and their implications on TME.

RNAi and Target Receptor	Model	Effects of Its Silencing	Delivery Strategy	Reference
siRNA PD-1	Cryopreserved human pan T cells/C57BL/6 mice	Decrease the expression of PD-1 on TCD4+ and TCD8+ surface/increase in IFN-γ and CXCL10/promoted leukocyte and T cell infiltration	Stabilizing modifications, cholesterol, and a single-stranded phosphorothioate tail	[150]
shRNA PD-1	K562-CD19/PC3-PSCA/NOD-SCID-IL-2Rγ−/−(NSG) mice	Increase in CAR-T cell proliferation, anti-tumor efficiency, and cytokine release in vitro/reduced tumor volume and prolonged survival time in vivo	Transduction	[143]
siRNA CTLA-4	T cells originated from splenocytes from female C57BL/6 and C57BL/6-Tg (TcraTcrb)425Cbn/Crl (OT-II) mice	Increased proliferation of TCD8+/Enhanced activation of T cells and proliferation/decrease in regulatory T cell/reduced tumor volume and prolonged survival time in vivo	PEG–PLA copolymer with cationic lipid BHEM-Chol	[153]
siRNA CTLA-4 and siRNA PD-L1	HCT116 cells/Female C57BL6J mice	Inhibition of cell proliferation and enhanced cell apoptosis/enhanced activation of T cells/upregulation of IFN-γ, IL-2, and TNF-α/reduced tumor volume and prolonged survival time in vivo	Exosome	[154]
shRNA CCR1 and shRNA CCR5	CT1, CT26, TSA, MCA203, DA3, A20, B16Lu8, HEK293, MDA-MB-231, cell lines/BALB/c, C57BL/6J, and NSG mice	Modification in the phenotype and function of polymorphonuclear cells, macrophages, and DCs/decrease in tumor growth and metastasis	Dendrimer-based nanoparticles (4PD NPs)	[155]
siRNA CXCR4	C57BL/6 mice with B16-F10 cells	Downregulation of genes involved in cell survival, cell adhesion, invasion and angiogenesis. Decrease in tumor growth and weight	-	[156]
miR-101 and siRNA CXCR7	4T1-luc2-M cells/BALBc mice	Inhibited cell proliferation, induced G1 arrest, increased apoptosis and suppressed invasion in vivo; inhibited STAT3 phosphorylation and the expression of STAT3 target genes; inhibited tumor growth and lung metastasis; and induced BrC apoptosis	-	[157]
shRNA-A2bR	769-P and Caki-1 cells/BABL/c mice	Inhibited the proliferation and migration in vitro/inhibited tumor growth in vivo/suppression of MAPK/JNK pathway	-	[158]
shRNA A2bR	OVCAR3, Hey, SKOV3, IGROV1 and PA1 cell lines	Suppression of IL-6-STAT3 signaling/overcame the drug resistance	-	[159]
siRNA VEGFR-1	M14-VR cells	Increases sensitivity to the BRAFi/inhibited invasive profile	-	[160]
siRNA VEGFR-3	MDA-MB-231, MDA-MB-468, and MCF7 BC cells	Decrease in cell proliferation and migration	-	[161]
shRNA HER3	MCF-7 and ZR75-1 cells	Increase in DNA damage through γH2AX/enhanced the radiosensitivity of luminal A breast cancer cells in vivo	-	[162]
siRNA VEGFR2 e siRNA EGFR	A549 cells and BALB/C mice	Inhibited tumor growth and side effects in vivo/prolonged survival time in vivo	PEI NPs	[163]
siRNA IGF1R	4T1 and CT26 cells	Higher apoptosis rate/cell cycle blockage/inhibited growth and metastasis	NPs loaded with phycoerythrin (PE)-conjugated siRNAs	[164]
siRNA IL-10A	TC-1 cells/C57BL/6 mice	Higher E7-specific IFN-γ+ CD8+ T cell precursors/inhibition of ERK and increase in the BIM protein (pro-apoptosis molecule)/shift from Th2 to Th1	-	[165]
siRNA IL-1R8	K562, Karpas 299, Nalm-18 and IMR-32 cell line	Dysregulation of NK receptors, cytokines, chemokines, and other factors	-	[166]
shRNA TLR4	U-87MG cell line	Increase in cell apoptosis/inhibited cell proliferation	-	[167]
shRNA TLR4	Nude mouse xenograft model	Lower expression of iNOS, MIP-3*α* and VEGF	-	[168]
siRNA SRA	C57BL/6 mice	Increase the immunogenicity of DCs, higher cytolytic activity by T cells/higher secretion of IFN-γ, growth inhibition, and better mice survival	Chitosan-siRNA complex	[169]

No delivery systems used (-).

In parallel to studies with PD-1, studies aiming to silence the CTLA-4 show an excellent alternative for restoring the antitumor response. S.-Y. Li et al. (2016) evaluated siRNA-CTLA-4 in vitro and in the murine model and observed its role in modulating T cell functionality [153]. They observed a decrease in the receptor expression and an increase in the inflammatory response to contribute to the tumor combat (Table 4) [153]. Furthermore, in this study, siRNA-CTLA-4 was delivered through polymeric particles (PEG–PLA copolymer with cationic lipid BHEM-Chol), leading to a target delivery and increasing the efficacy of this therapy [153].

In addition to silencing unique targets, an interesting strategy is to silence more than one target simultaneously. In this context, J. Li et al., 2023, evaluated the silencing of the CTLA-4 receptor and the PD-L in colorectal cancer, observing, in vitro, a reduction in cell proliferation and a higher rate of apoptosis [154]. In vivo, a diminution in tumor growth, an increase in CD8+ T cells at the tumor site and in the secretion of interleukin 2 (IL-2), TNF-α, and IFN-γ were observed (Table 4) [154]. In this study, exosomes were used to carry the siRNA, showing satisfactory results, especially when carrying both siRNAs compared to just one [154].

### 4.2. G Protein-Coupled Receptors Silencing

#### 4.2.1. Chemokine Receptor Silencing

G protein-coupled receptors (GPCRs) participate in the processes of communication with extracellular molecules and signal transduction [170]. Moreover, they are targets of inflammatory mediators, promoting the connection between chronic inflammation and cancer [171]. These receptors, highly expressed in different types of tumors, are involved in cellular regulation processes for tumor growth [172]. Their structure features an extracellular domain, a transmembrane region with seven loops, an intracellular region, and a terminal tail [173]. The activation of this receptor can occur through various ligands, such as chemokines, neurotransmitters, hormones, and peptides [174].

The GPCR family perform various biological functions and can be classified as constitutive or inflammatory receptors [175]. Chemokines are primarily known for their role in stimulating cell migration [176]. The binding of chemokines with their receptors can be involved in angiogenesis, metastasis, proliferation, and leukocyte recruitment [171].

Chemokine receptors, such as C-C chemokine receptor type 1 (CCR1), C-X-C chemokine receptor type 4 (CXCR4) and C-C chemokine receptor type 5 (CCR5) are highly expressed in different types of cancers [155]. These receptors, when activated by their ligands, promote tumor development through the recruitment of TAMs, accumulation of MDSCs, and differentiation of myeloid cells [155,177]. In the study by Zilio et al. (2022), the silencing of CCR1 and CCR5 through shRNA modulates the TME, leading to an antitumor response (Table 4) [155]. In this study, dendrimer-based nanoparticles (4PD NPs) were used for delivery, targeting myeloid cells infiltrated in the tumor. In their in vivo experiments, a decrease in tumor growth and metastasis was observed, and this also allowed for a modification in the phenotype of TME cells, favoring the anti-tumoral process and a decrease in the production of MDSCs [155].

Regarding CXCR4, its overexpression is related to an increase in cell proliferation, migration, and invasion, while knockdown led to a decrease in these rates [178]. Interestingly, a relationship with HIF-a was observed, and its silencing through shRNA led to a decrease in receptor expression [179]. In the study by X.Y. Tan et al. (2014), the silencing of CXCR4 led to a decrease in cell proliferation of the QBC939 cholangiocarcinoma cell line [178]. Furthermore, in co-culture with perineural cells, silencing reduced neuronal invasion, which is associated with a worse prognosis of this disease (Table 4) [178].

Another study used shRNA-CXCR4 to evaluate its role in vitro and in vivo in melanoma, and observed a decrease in mRNA and protein levels, in B16-F10 cells, and an impairment in the tumor growth in the treated mice [156]. An important aspect to pay attention to in this study is the construction of two shRNA-CXCR4, with different effectiveness, indicating that the region of the target mRNA is an important aspect to be considered in the design of the RNAi.

Another chemokine receptor, C-X-C chemokine receptor type 7 (CXCR7), is also highly expressed in different types of cancers and involved in biological processes that favor the carcinogenesis. J.T. Li et al. (2015) discovered that miRNA-101 has a role in the regulation of the carcinogenic process of breast cancer [157]. Tumor samples from patients were collected and studied, revealing a low expression of miRNA-101, primarily in more advanced tumors (Table 4) [157]. This study also looked at the relationship between miRNA-101 and CXCR7 and found that when miRNA-101 was present, receptor expression decreased [157]. Furthermore, siRNA-mediated silencing CXCR7 resulted in decreased cell proliferation, increased apoptosis, and decreased invasion, indicating the receptor’s pro-tumoral role and relationship with miRNA-101 [157].

#### 4.2.2. Adenosine Receptor Silencing

Adenosine is an important intermediate in the synthesis of adenosine triphosphate (ATP) participates in metabolic processes and acts as an inhibitor of the immune response [180,181]. In tumors, there are large amounts of extracellular adenosine, where the tumor benefits from an immunosuppressed environment [180]. From the interaction with its receptors (A1aR, A2AaR, A2baR, and A3aR), adenosine is able to modulate the TME, favoring carcinogenesis [182].

A study sought to silence the A2aR in renal cell carcinoma. In in vitro experiments, a reduction in cell proliferation, as well as a decrease in migration and invasion, was observed (Table 4) [158]. Interestingly, the addition of a receptor agonist was not able to reverse the effect of shRNA silencing. However, in cells treated with an antagonist, the addition of the agonist restored the pro-tumor environment [158]. Similar results were observed in vivo, with the reduction in tumor growth in the mice [158].

It was detected that in ovarian tumors resistant to the drug Olaparib, there is high dose-dependent expression of A2bR [160]. It was observed, through silencing with siRNA, that adenosine has a relationship with the expression of IL-6, an inflammatory mediator (Table 4) [159]. The cells that had low receptor expression also showed low interleukin expression [159]. Similarly, its relationship with Signal transducer and activator of transcription 3 (STAT3) was indicated, implying the connection of the IL-6/STAT3 signaling pathway with A2bR [159]. It was also identified that treatment with siRNA, a STAT3 inhibitor, and Olaparib showed promising results for overcoming drug resistance [159].

### 4.3. Growth Factor Receptors Silencing

Growth factor receptors are part of the receptor tyrosine kinase (RTK) family, which are important for the signal transduction of various processes [183]. Its structure features an extracellular, a transmembrane, and an intracellular domain, followed by the tyrosine kinase domain and a terminal tail [184]. The interaction of the receptor with its ligand promotes the increase in tyrosine kinase receptor activity and the autophosphorylation of the tyrosine residue, activating a signaling cascade [185,186]. The increased expression of RTKs, the vascular endothelial growth factor receptor (VEGFR) and the endothelial growth factor receptor (EGFR), is present in different types of cancers [185]. In cancer, the aberrant activation of these receptors occurs, whether through their overexpression, mutation, chromosomal translocation, or autocrine activation [183].

VEGFR is one of the most studied receptors, and together with its ligand VEGF, it plays an important role in the angiogenic process and, consequently, in tumor vascularization [187]. This receptor can be differentiated into VEGFR-1, VEGFR-2, and VEGFR-3, with VEGFR-1 being essential for hematopoiesis, MMP activation, and the migration of immune cells to the TME, while VEGFR-2 plays a role in vasculogenesis VEGFR and angiogenesis, and VEGFR-3 is mainly associated with lymphangiogenesis [188,189].

EGFR is also an important receptor for cell signaling, and its overexpression or mutation contributes to carcinogenesis [190]. Its overexpression can lead to the activation of various signaling pathways, which promote cell proliferation, metastasis, and angiogenesis [190]. The mutation of this receptor is associated with an increase in the immunosuppressive profile of the TME through the upregulation of PD-1 [191].

Among the studies on the silencing of growth factors, the silencing of VEGFR-1 through siRNA led to an improvement in resistance to BRAF inhibitors (BRAFi) (Table 4) [160]. In patients with melanoma, their response is inactivated by acquired resistance, due to the elevation of the VEGF-A and VEGF-1 concentrations, favoring tumor growth [160]. Thus, through the blockade of this signaling pathway, there was a lower drug resistance in melanoma-infected cells, thereby reducing the invasive profile, highlighting the importance of this receptor and its silencing in melanoma patients [160].

The same effect was monitored with VEGFR-3 in breast cancer, where its high expression was detected in cells that were resistant to doxorubicin (Table 4) [161]. In this study, VEGFR-3 silencing was performed using siRNA in parental and resistant cells. The results showed a decrease in cell proliferation and migration, mainly in cells that were not resistant to the drug [161]. Despite the absence of evidence in resistant cells, the study allowed for the observation that high VEGFR-3 expression is related to a worse prognosis and lower efficacy of doxorubicin [161].

Another well-studied growth factor is EGFR, also known as human epidermal growth factor receptor (HER). In the study by He et al. (2018), a breast cancer cell line with low HER3 expression was developed using shRNA-HER3 to observe its effect on radiosensitivity (Table 4) [162]. Thus, it was observed that HER3 silencing, in addition to decreasing cell proliferation and increasing apoptosis, improves the efficacy of radiotherapies [162]. This improvement in efficacy is due to the increased DNA damage caused by radiation and the decrease in repair [162].

To explore the efficacy of combinatorial silencing, researchers evaluated the simultaneous blockade of VEGFR and EGFR in adenocarcinoma cell lines [192]. The siRNA was carried from positively charged PEI-siRNA, which, due to its cationic charge, protects the RNAi from degradation. The results indicated that, although silencing either receptor individually effectively inhibited tumor growth, dual silencing of both receptors produced a significantly superior outcome (Table 4) [163,193]. On the other hand, the administration of a double dose of a single siRNA was less effective than the combined therapy, highlighting the importance of targeting both pathways. Furthermore, the combination of dual receptor silencing with the chemotherapeutic agent cisplatin produced the most potent antitumor effect [163].

In the study by Abolhasani et al. (2023), siRNA-IGFR was used to silence the insulin-like growth factor (IGFR), which is involved in proliferation and metastasis [164,194]. In this study, siRNA-HIFa was also used to silence HIFa, with the aim of modulating the immunosuppressive environment (Table 4) [164]. Both siRNAs were delivered from NPs loaded with phycoerythrin (PE)-conjugated siRNAs. It was observed that the combined therapy was more effective in modulating the TME than therapy with only one of the siRNAs in breast and colon cancer cells [164].

### 4.4. Interleukin Receptors Silencing

Interleukins are proteins of the immune system that participate in the initiation and modulation of the inflammatory response [195]. They can be divided into inflammatory interleukins, which participate in the activation of cells to combat infectious agents, and anti-inflammatory interleukins, which play a role in controlling the activation of the immune response and promoting tissue repair [196]. In carcinogenesis, interleukins can participate in processes that favor growth and metastasis [197].

Aiming to improve the response of DCs, Ahn et al. (2015) sought to silence various molecules, such as cyclooxygenase-2 (COX-2), mothers against decapentaplegic homolog 2 and 3 (SMAD2 and SMAD3), IL-10, interleukin 10 receptor subunit alpha (IL-10RA), inducible nitric-oxide synthase (iNOS), interleukin-1 receptor associated kinase 3 (IRAK3), suppressor of cytokine signaling 1 (SOCS-1), Src homology 2-containing inositol 5-phosphatase 1 (SHIP-1), TGF-β, or transforming growth factor beta (TGFβ) receptors (TGF-βR) [165]. This study was performed in the TC-1 P0 tumor model, a cervical cancer model that expresses HPV16 E7 protein, and also in the TC-1 (P3), an immune-resistant tumor model. In the experiments, it was observed that the silencing of IL-10RA and TGF-βR resulted in a more pronounced CD8+ T cell response, specific to the HPV E7 protein, and a heightened antitumor response [165]. In addition, comparisons were made with previous studies where the silencing of the Bcl-2-interacting mediator of cell death (BIM) and phosphatase and tensin homolog (PTEN) was performed [198]. Various combinations were evaluated, specifically siIL-10RA, siIL-10RA + siPTEN, siIL-10RA + siBIM, and siIL-10RA + TGFBR. It was determined that the concurrent silencing of both receptors was more effective in eliciting TDC8+ responses and enhancing DC resistance to apoptosis (Table 4) [165].

Landolina et al. (2022) transfected six siRNAs targeting Interleukin-1 receptor 8 (IL-1R8) into NK cells to elucidate the receptor’s role in the tumor microenvironment [166]. It was also observed that the silencing of IL-1R8 dysregulated different cellular pathways due to the dysregulation of NK receptors, cytokines, chemokines, and other factors (Table 4) [166]. The decrease in receptor expression also affected the functionality of NKs, resulting in a reduction in cytotoxic activity [166].

### 4.5. Pattern Recognition Receptors (PRRs) Silencing

There are also PRRs, exemplified by Toll-like receptors (TLR), that interact with their ligand and activate a signaling cascade that primarily activates the innate immune response through the activation of APCs [199]. This also activates cytokines, which interact with T cells to modulate an adaptive response, generating immunity [199]. These receptors are involved in angiogenesis, metastasis, and immune evasion, as well as anti-tumor processes, through the induction of apoptosis [200].

Among the TLRs, one of the most studied is TLR4, which plays an important role in neoplastic development, although other TLRs are also involved in different types of cancers [200,201]. This receptor is involved in the angiogenesis, maturation and activation of dendritic cells, and infiltration of M2 macrophages [202]. Thus, Y. Liu, et al. (2020) used shRNA with the aim of silencing TLR4 to understand the mechanisms behind its effects on glioma cells (Table 4) [167]. Different shRNA sequences were also constructed, and the one that achieved the best silencing result was chosen for future experiments [167]. It was observed that in addition to the decrease in expression, there was an inhibition of growth and induction of apoptosis [167]. Although it does not address aspects of the TME, the receptor’s role in mediating innate immunity and its connections to the STAT3 and AKT/GSK3β/β-catenin pathways suggest that silencing this receptor could potentially influence TME modulation [203,204].

Still regarding TLR4, N. Jiang et al. (2020) sought to understand its role in cervical cancer through silencing it with shRNA [168]. It was observed that the receptor is associated with the expression of various pro-inflammatory cytokine molecules (Table 4) [168]. It was observed that with low receptor expression due to its silencing, there was less tumor growth compared to its overexpression, both in vitro and in vivo [168]. Along with these results, the receptor’s relationship with iNOS, macrophage inflammatory protein-3 alpha (MIP-3α), and VEGF was explored. Silencing the receptor resulted in decreased secretion of these mediators, which are responsible for the induction of metastasis, angiogenesis, and the recruitment of immunosuppressive cells to the microenvironment.

In addition to TLRs, there are scavenger receptors, which are highly expressed in myeloid-derived cells, including DCs and macrophages, playing an important role as immunosuppressors, mainly of DCs [169]. The silencing of Scavenger Receptor A (SRA) has been studied to improve the functioning of DCs, helping to enhance the efficacy of chaperone vaccines in melanoma patients (Table 4) [169]. Studies show that chaperone vaccines are recognized by APCs, mainly DCs, so the inhibitory role of SRA in DCs can hinder the action of this vaccine in patients. In the study by Qian et al. (2023), it was observed that silencing this receptor through shRNA carried by an adjuvant, chitosan, can increase the ability of DCs to capture the vaccine and enhance the antitumor response [169]. In addition to increasing the secretion of IFN-y and the activity of cytotoxic T lymphocytes (CTLs), this optimized the efficacy of the chaperone vaccine [169].

All these studies point to the importance of receptors in the TME, as well as in tumor progression. Furthermore, they demonstrate not only the role of RNAi in silencing a receptor with the aim of modulating its pro-tumor response, but also its potential for studying the role of a specific receptor in the TME. Thus, it is possible to infer the importance of silencing receptors, modulating cell communication, and, consequently, TME.

## 5. Future Perspectives

Despite the promising results of receptor silencing in reversing proliferation, cell invasion, and tumor growth, some of the studies found do not directly discuss the change in the cellular profile of the TME. Although it is possible to infer a change to an antitumor profile, it is unclear how these changes occurred and how they affect the TME. The change, for example, in the secretion profile of cytokines and chemokines observed in most studies may alter the cellular profile of the TME and contribute to the reversal of carcinogenesis.

Regarding delivery strategies, more studies are needed to ensure specific delivery, better stability, and better results. Products that are already approved by the FDA have chemical modifications and are conjugated with other molecules, with the aim of improving pharmacokinetics and pharmacodynamics. Other platforms, like NPs, are also being developed; however, one of the limitations that is still encountered is ensuring delivery to hard-to-reach locations, such as solid tumors.

In addition to using delivery platforms to improve efficiency and expected results, another way to increase the generated antitumor response is to use RNAi in conjunction with other therapies, as monoclonal antibodies. As seen in this study, when using a second therapeutic strategy, better results were observed compared to therapies used in isolation. It is also possible to silence more than one gene, obtaining promising results, as observed in the results. However, further studies are needed to ensure the safety of this strategy, as well as to determine how many genes can be silenced while guaranteeing patient safety.

## 6. Conclusions

Despite the promising results, RNAi still requires delivery platforms to overcome its challenges, such as degradation by enzymes and low delivery capacity. In this study, it was observed that the use of nanoparticles and microorganisms as carriers further increases the efficacy of RNAi and favors its delivery to specific cells. Therefore, we need to conduct additional research to overcome these constraints. RNAi proves to be an effective and innovative tool for gene silencing to modulate the TME and regulate the immune response, as seen with various approved and developing products. Through this study, it was observed that the modulation of receptor expression proves to be an intriguing alternative, both to reduce the anti-tumor response and to study how the receptor acts in the tumor. In addition to being an intriguing therapeutic strategy when used as a single approach, silencing can increase the efficacy of the therapy when combined with other therapies, such as silencing other molecules and different immunotherapy approaches, but can also overcome the limitations and resistance generated by the tumor and due to the heterogeneity of the TME. Additional research is required to examine the TME across various cancers and its role in carcinogenesis. Furthermore, investigations into delivery platforms that facilitate more precise targeting are essential to improve efficacy and minimize adverse effects.

## Figures and Tables

**Table 1:** Drugs, already approved and under study, that use RNAi as a silencing strategy (until January 2026).

Drug	Development Phase	Target Disease	Target Gene	Reference
Patisiran (Onapattro)	Approved (2018)	Hereditary transthyretin amyloidosis	TTR	NCT01617967
Givosiran (Givlaari)	Approved (2019)	Acute hepatic porphyria	ALAS1	NCT02949830
Lumasiran (Oxlumo)	Approved (2020)	Primary hyperoxaluria type 1	HAO1	NCT04125472
Inclisiran (Laqvio)	Approved (2021)	Primary hyperlipidemia	PCSK9	NCT04873934
Vutrisiran (Amvuttra)	Approved (2022)	Hereditary transthyretin amyloidosis	TTR	NCT03759379
Nedosiran (Rivfloza)	Approved (2023)	Primary hyperoxaluria type 1	LDHA	NCT05993416
Fitusiran (Qfitlia)	Approved (2025)	Hemophilia A or B	SERPINC1	NCT07285460
Plozasiran (Redemplo)	Approved (2025)	Familial chylomicronemia syndrome	APOC3	NCT06880770
ADX-324	Phase III	Hereditary angioedema	KLKB1	NCT06960213
STP705	Phase II	Skin squamous cell carcinoma in situ	TGFB1/PTGS2	NCT04844983
siG12D LODER	Phase II	Pancreatic ductal adenocarcinoma	KRAS G12D	NCT01676259
ALN-CFB	Phase I/II	Paroxysmal nocturnal hemoglobinuria	CFB	NCT07187401
STP707	Phase I	Solid tumors (refractory patients to standard therapy)	TGFB1/PTGS2	NCT05037149
siRNA-EphA2-DOPC	Phase I	Advanced or recurrent solid tumors	EPHA2	NCT01591356
PDR001	Phase I	Parkinson’s disease	SNCA	NCT07157345

Transthyretin (TTR), 5′-Aminolevulinate Synthase 1 (ALAS1), hydroxyacid oxidase 1 (HAO1), proprotein convertase subtilisin/kexin type 9 (PCSK9), lactato desidrogenase A (LDHA), serpin family C member 1 (SERPINC1), apoplipoprotein C3 (APOC3), kallikrein B1 (KLKB1), transforming growth factor beta 1 (TGFB1), prostaglandin-endoperoxide synthase 2 (PTGS2), kirsten rat sarcoma viral oncogene homolog mutation (KRAS G12D), complement factor B (CFB), ephrin type-A receptor 2 (EPHA2), and synuclein alpha (SNCA).

**Table 2:** Effects of tumor microenvironment cellular components on cancer.

Cellular Component	Alteration in Cancer	Effect of the Alteration	Reference
Cancer-Associated Fibroblasts (CAFs)	Increased activation and phenotypic heterogeneity (pro-tumoral subtypes predominate)	Secretion of cytokines and growth factors (TGF-β, CXCLs), remodeling of the extracellular matrix (ECM), and induction of angiogenesis, invasion, and immunosuppression	[100,101]
Endothelial Cells (ECs)	Overexpression of VEGF and VEGFR; abnormal and dysfunctional vessels	Promotes hypoxia, metastasis, and therapeutic resistance; modulation of tumor perfusion influences response to immunotherapies	[102]
Tumor-Associated Macrophages (TAMs)	Increased infiltration and M2 (immunosuppressive) polarization	Stimulates angiogenesis, secretion of MMPs, suppression of T lymphocytes, and promotion of metastasis	[103,104]
Myeloid-Derived Suppressor Cells (MDSCs)	Systemic and intratumoral expansion	Inhibition of T and NK cells via arginase, iNOS, and ROS; reduces the efficacy of immunotherapies	[105]
Tumor-Associated Neutrophils (TANs)	Increased recruitment and phenotypic shift (N1 → N2)	Release of proteases and NETs, promoting invasion, metastasis, and immunosuppression	[106]
Dendritic Cells (DCs)	Reduced maturation and antigen-presenting capacity	Induces immune tolerance and limits adaptive immune response	[102]
T Lymphocytes (CD8+, CD4+) and regulatory lymphocytes (Treg)	CD8+ exhaustion (↑ PD-1) and accumulation of Treg in the TME	Suppression of cytotoxicity and immune escape; reversible with checkpoint blockade (PD-1/PD-L1)	[107]

Transforming growth factor beta 1 (TGF-β), C-X-C motif chemokine ligands (CXCLs), vascular endothelial growth factor (VEGF), vascular endothelial growth factor receptor (VEGFR), M2 macrophages (M2), matrix metalloproteinases (MMPs), natural killers (NK), inducible nitric-oxide synthase (iNOS), reactive oxygen species (ROS), N1 neutrophils (N1), N2 neutrophils (N2), neutrophil extracellular traps (NETs), programmed cell death 1 (PD-1), programmed cell death 1 ligand (PD-L1) and Increase (↑).

## Data Availability

Not applicable.

## Abbreviations

A2aR	Adenosine A2A receptor
A2bR	Adenosine A2B receptor
AGO	Argonaute protein
ALAS1	5′-Aminolevulinate Synthase 1
APC	Antigen-presenting cell
ATP	Adenosine Triphosphate
ATTRv	Hereditary transthyretin amyloidosis
BLC2	B-Cell Lymphoma 2
BIM	Bcl-2 Interacting Mediator of cell death
BRAFi	BRAF inhibitors
CAFs	Cancer-associated fibroblasts
CAR	Chimeric antigen receptor
CCR1	CC chemokine receptor type 1
CCR5	CC chemokine receptor type 5
CD4+ T	Helper T lymphocyte
CD8+ T	Cytotoxic T lymphocyte
CEL	Cationic cholesterol derivative
CFB	Complement Factor B
coRNAi	Combinatorial RNAi
COX-2	Cyclooxygenase-2
CTL	Cytotoxic T-lymphocyte
CTLA-4	Cytotoxic T-lymphocyte antigen 4
CXCL10	C-X-C motif chemokine ligand 10
CXCLs	C-X-C motif chemokine ligands
CXCR4	C-X-C chemokine receptor type 4
CXCR7	C-X-C chemokine receptor type 7
DC	Dendritic cell
DOPE	Dioleoylphosphatidylethanolamine
DOTAP	1,2-dioleoyl-3-trimethylammonium-propane
DOX	Doxorubicin
ECM	Extracellular matrix
ECs	Endothelial cells
EGF	Epidermal growth factor
EGFR	Epidermal growth factor receptor
EPHA2	Ephrin Type-A Receptor 2
FDA	Food and Drug Administration
GPCRs	G protein-coupled receptors
HAO1	Hydroxyacid oxidase 1
HER	Human epidermal growth factor
HIF	Hypoxia-inducible factor
IFN-y	Interferon gamma
IGFR	Insulin-like growth factor
IL-10	Interleukin 10
IL-10RA	Interleukin 10 receptor subunit alpha
IL-12	Interleukin 12
IL-6	Interleukin 6
iNOS	Inducible nitric-oxide synthase
IRAK3	Interleukin-1 receptor associated kinase 3
KLKB1	Kallikrein B1
KRAS G12D	Kirsten Rat Sarcoma Viral Oncogene Homolog Mutation
LDHA	Lactato Desidrogenase A
LNPs	Lipid nanoparticles
M1	M1 macrophages
M2	M2 macrophages
MIP-3α	Macrophage inflammatory protein-3 alpha
miRNA	Micro RNA
mMDSCs	Myeloid-derived suppressor cell
MMPs	Matrix metalloproteinases
MON	Mesoporous organosilica nanoparticle
mRNA	Messenger RNA
N1	N1 neutrophils
N2	N2 neutrophils
NETs	Neutrophil extracellular traps
NK	Natural killers
PCSK9	Proprotein convertase subtilisin/kexin type 9
PD-1	Programmed cell death protein 1
PD-L1	Programmed cell death protein 1 ligand
PE	Phycoerythrin
PEI	Polyethyleneimine
PLGA	Poly(lactic-co-glycolic acid)
Pre-shRNA	Short hairpin precursor
PRRs	Pattern recognition receptors
PS	Phosphororhioate
PTEN	Phosphatase and tensin homolog
PTGS2	Prostaglandin-Endoperoxide Synthase 2
PTX	Paclitaxel
RISC	RNA-induced silencing complex
RNAi	RNA interference
ROS	Reactive oxygen species
RTK	Receptor tyrosine kinase
SHIP-1	Src homology 2-containing inositol 5-phosphatase 1
shRNA	Short hairpin RNA
siRNA	Small interfering RNA
SMAD2	Mothers against decapentaplegic homolog 2
SMAD3	Mothers against decapentaplegic homolog 3
SNCA	Synuclein Alpha
SOCS-1	Suppressor of cytokine signaling 1
SRA	Scavenger receptor A
STAT3	Signal Transducer And Activator Of Transcription 3
TANs	Tumor-associated neutrophils
TGFB1	Transforming Growth Factor Beta 1
TGF-β	Transforming Growth Factor-beta 1
TGF-βR	Transforming Growth Factor-beta 1 receptor
TLR	Toll like receptor
TLR4	Toll-like receptor 4
TME	Tumor microenvironment
TNF-α	Tumor necrosis factor alfa
Treg	Regulatory T cells
TTR	Transthyretin
VEGF	Vascular endothelial growth factor
VEGF-1	Vascular endothelial growth factor-1
VEGF-A	Vascular endothelial growth factor-A
VEGFR	Vascular endothelial growth factor receptor
VEGFR-1	Vascular endothelial growth factor receptor-1
VEGFR-3	Vascular endothelial growth factor receptor-3
